# Case Report: Novel compound heterozygous *TPRKB* variants cause Galloway-Mowat syndrome

**DOI:** 10.3389/fped.2024.1360867

**Published:** 2024-04-03

**Authors:** Takuya Hiraide, Taiju Hayashi, Yusuke Ito, Rei Urushibata, Hiroshi Uchida, Ryoichi Kitagata, Hidetoshi Ishigaki, Tsutomu Ogata, Hirotomo Saitsu, Tokiko Fukuda

**Affiliations:** ^1^ Department of Pediatrics, Hamamatsu University School of Medicine, Hamamatsu, Japan; ^2^ Department of Biochemistry, Hamamatsu University School of Medicine, Hamamatsu, Japan; ^3^ Department of Pediatrics, Hamamatsu Medical Center, Hamamatsu, Japan; ^4^ Department of Hamamatsu Child Health and Development, Hamamatsu University School of Medicine, Hamamatsu, Japan

**Keywords:** *TPRKB*, Galloway-Mowat syndrome, exome sequencing, KEOPS complex, nephrotic proteinuria

## Abstract

**Background:**

Galloway-Mowat syndrome (GAMOS) is a rare genetic disease characterized by early-onset nephrotic syndrome and microcephaly with central nervous system abnormalities. Pathogenic variants in genes encoding kinase, endopeptidase, and other proteins of small size (KEOPS) complex subunits cause GAMOS. The subunit *TPRKB* (TP53RK binding protein) has been reported in only two patients with GAMOS with homozygous missense variants.

**Clinical report:**

Herein, we described a three-year-old male with GAMOS. He exhibited developmental delay, developmental regression, microcephaly, distinctive facial features, skeletal abnormalities, and epilepsy. Brain magnetic resonance imaging revealed progressive brain atrophy, delayed myelination, T2-hypointense signals in the thalamus, and multiple intracranial abnormal signals on diffusion-weighted imaging. He presented with relapsing nephrotic proteinuria exacerbated by upper respiratory tract infections and progressive renal function decline. Exome sequencing identified compound heterozygous missense and frameshift variants in *TPRKB*: c.224dup, p.(Ser76IlefsTer3) and c.247C>T, p.(Leu83Phe).

**Conclusions:**

Our study supports that pathogenic *TPRKB* variants cause KEOPS complex-related GAMOS.

## Introduction

Galloway-Mowat syndrome (GAMOS; MIM#251300) is a rare genetic disease characterized by early-onset nephrotic syndrome (NS), microcephaly, and brain anomalies that result in severely delayed psychomotor development (1). The renal prognosis of GAMOS is poor, and renal replacement therapy or renal transplantation is necessary for survival (2). GAMOS is clinically and genetically heterogeneous. Other clinical features include dysmorphic facial features, skeletal anomalies, and esophageal hiatal hernias. Several genes have been reported to be associated with GAMOS, including *WDR73*, *LAGE3*, *OSGEP*, *TP53RK*, *TPRKB*, *WDR4*, *NUP107*, *NUP133*, GON7, *YRDC*, and *PRDM15* (2–8).

*TPRKB* (TP53RK binding protein) (MIM*608680) encodes a subunit of the highly conserved kinase, endopeptidase, and other proteins of small size (KEOPS) complex. The human KEOPS complex comprises OSGEP, TP53RK, TPRKB, LAGE3, and GON7 (9). It regulates the universal chemical modification of tRNA, N^6^-threonylcarbamoyl adenosine (t^6^A), which is essential for normal cell growth and accurate translation by ribosomes (10). The KEOPS complex is involved in human podocyte migration through impaired cell proliferation, increased apoptosis, genomic instability, and defects in actin regulation (4). Biallelic pathogenic variants in genes of the KEOPS complex are responsible for GAMOS in many patients (3, 4). Renal biopsies of patients with KEOPS complex-related GAMOS reveals mainly focal segmental glomerulosclerosis or diffuse mesangial sclerosis, with partial podocyte foot process effacement on electron microscopy (4). In contrast, GAMOS 5 (MIM#617731) caused by *TPRKB* has only been reported in two patients with homozygous missense variants (4). Herein, we report the case of a patient with GAMOS with compound heterozygous missense and frameshift variants in *TPRKB*.

## Clinical report

**Figure 1:**
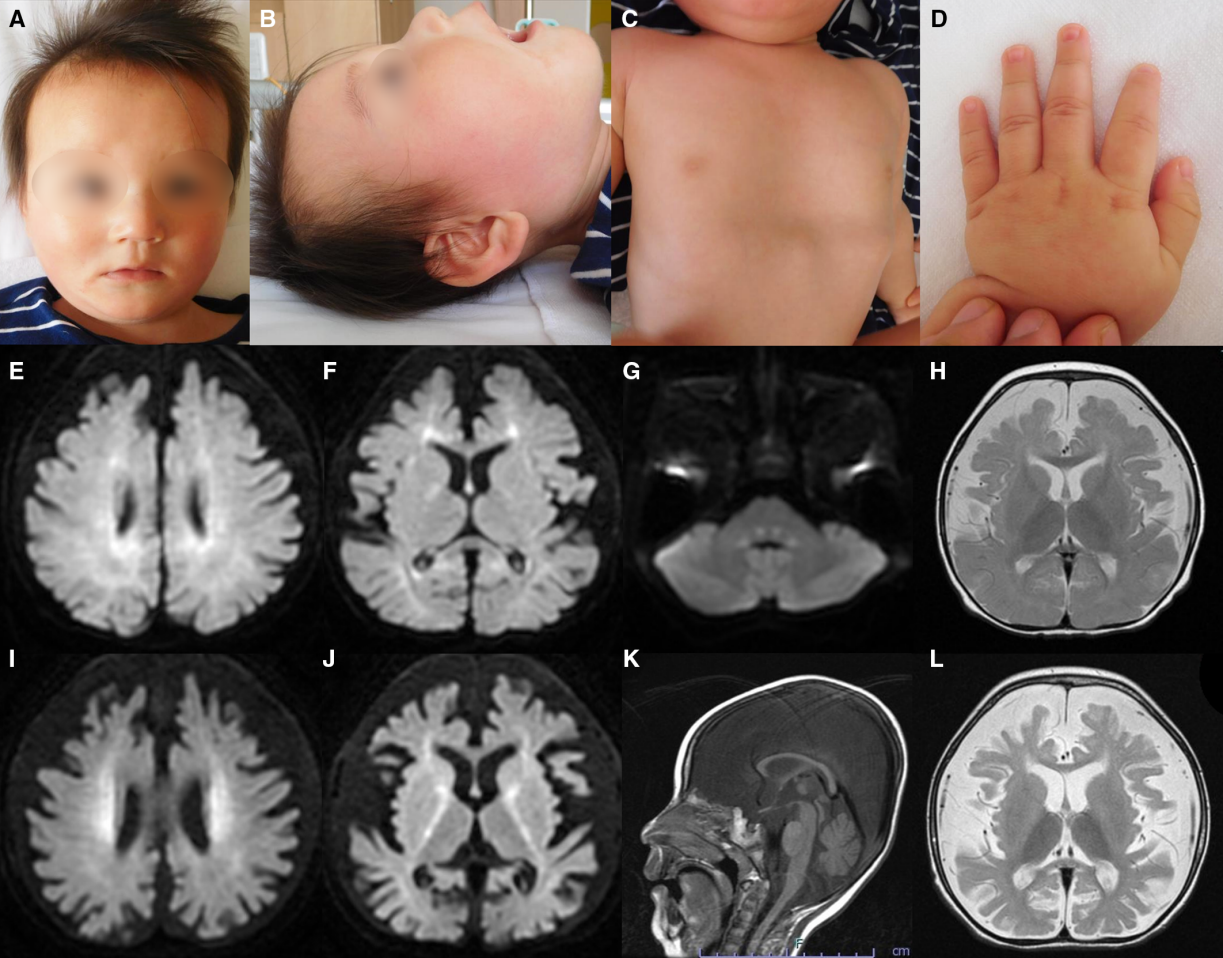
Representative clinical findings. (**A**–**D**) Photographs of the patient at ten months of age. Widely spaced eyes, epicanthus, left esotropia, medial sparse eyebrow, pointed chin, and thin upper lip vermilion are observed (**A**, **B**). Pectus excavatum (**C**) and tapered fingers (**D**) are recognized. (**E**–**L**) Brain magnetic resonance imaging (MRI) at ten months of age (**E**–**H**) and one year and eight months of age (**I**–**L**). Axial diffusion-weighted imaging (DWI) demonstrated elevated signal in the bilateral periventricular white matter, deep and subcortical white matter of the bilateral posterior lobe, posterior limb of the internal capsule, and dorsal part of the pons (**E**–**G**). An axial T2-weighted image shows brain atrophy with frontal white matter dominance and diffuse hypointense signal of the thalamus (**H**) DWI hyperintensities of the bilateral corona radiata and internal capsule are more distinct (**I**, **J**). A sagittal T1-weighted image shows atrophy of cerebellar vermis and thinning of the corpus callosum (**K**). An axial T2-weighted image reveals progressive brain atrophy and no hypointensity in the subcortical white matter. Hypointensity in the anterior limb of the internal capsule is obscured (**L**).

After 40 weeks of gestation without asphyxia, a Japanese boy was born to nonconsanguineous, healthy parents as their first child. His birth weight was 3,450 g [+1.04 standard deviation (SD)], his length was 51.0 cm (+0.90 SD), and his occipitofrontal circumference was 35.0 cm (+1.25 SD). Distinctive facial features such as widely spaced eyes and pointed chin, pectus excavatum, tapered fingers, esotropia, and muscle hypotonia, were noted (Figures 1A–D). He could hold his head up at five months of age and sit with support at eight months of age. At nine months of age, he babbled but could not maintain a sitting position, even with support. At one year and one month of age, antiepileptic drugs were initiated because of the onset of epileptic spasms and focal to bilateral tonic-clonic seizures. His development regressed, losing interest in toys at one year and three months of age and becoming unable to hold his head up at one year and eight months of age. Tube feeding was initiated because of dysphagia and weight loss at two years and one month of age, followed by gastrostomy at three years of age.

He was diagnosed with proteinuria at six months of age. The spot urine protein/creatinine ratio (UPCR) was 3.33 g/g at one year and one month of age. At 2 years and eight months of age, he was diagnosed with NS due to increased proteinuria (UPCR 17.23 g/g) and hypoalbuminemia (Alb 2.9 g/dl) induced by an acute upper respiratory tract infection. As the infection improved, the proteinuria decreased (UPCR 0.97 g/g). He initiated treatment with valsartan. Subsequently, NS relapsed during respiratory infections, with serum albumin ranging from 2.2 to 3.9 g/dl and UPCR ranging from 0.73 to 17.23 g/g. Serum creatine was low (0.15 mg/dl), while the cystatin C value, which was 0.46 mg/L at 2 years of age, increased to 0.66 mg/L at 3 years of age, suggesting a progressive decline in renal function. Renal biopsy was not performed because of respiratory depression during anesthesia.

Metabolic screenings of blood and cerebrospinal fluid, including amino acids, lactic acid, and pyruvic acid, were unremarkable. Interictal electroencephalogram at one year and eight months of age revealed multifocal spikes with slow background activity. Bilateral visual evoked potentials were absent. Electrocardiography, auditory brainstem response, nerve conduction studies, electroretinography, and echocardiography findings were normal. Radiography revealed no bone abnormalities other than scoliosis and pectus excavatum. Abdominal computed tomography scans of the kidneys, bladder, liver, and spleen revealed no abnormalities. Brain magnetic resonance imaging (MRI) showed a diffuse T2-hypointense signal in the thalamus and numerous characteristic aberrant DWI signals in the white matter, internal capsule, and dorsal part of the pons (Figures 1E–J, L). At one year and eight months of age, there was no T2-hypointensity in the subcortical white matter, suggesting hypomyelination (Figure 1L). The T2-hypointensity in the anterior limb of the internal capsule observed at 10 months of age was indistinct at one year and eight months of age, suggesting demyelination (Figures 1H, L). Progressive atrophy of the cerebral hemispheres and cerebellum was observed (Figures 1K, L).

**Figure 2:**
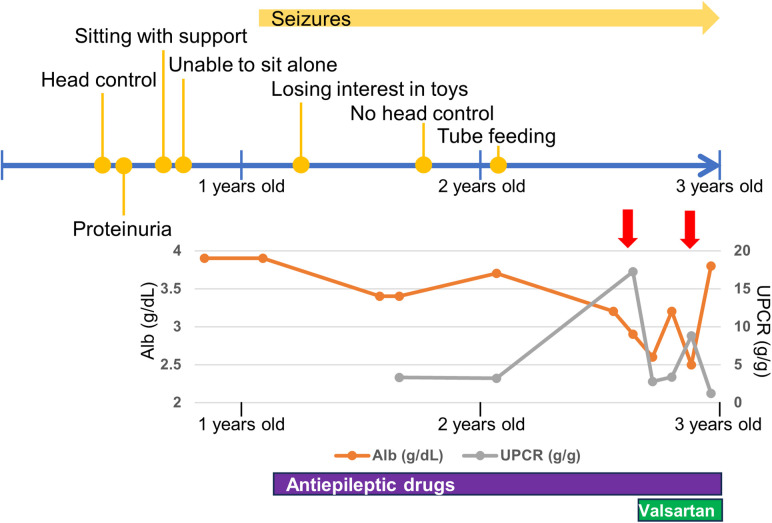
Timeline of neurological and renal symptoms. Red allows indicate respiratory infections. Alb, serum albumin; UPCR, spot urine protein/creatinine ratio.

The last physical examination at three years of age showed a body weight of 12.0 kg (−1.1 SD), height of 97.0 cm (+1.1 SD), and head circumference of 42.5 cm (−4.4 SD). He had a social smile, but his visual tracking was poor. He had constant stridor. Hypotonia of the upper limbs and trunk and spasticity of the lower limbs were noted. His deep tendon reflexes were hyperactive with lower-extremity dominance, and the Babinski reflex and ankle clonus were observed. These findings were suggestive of GAMOS. Progression of neuronal symptoms and repeated worsening of urinary proteinuria due to infectious disease are shown along the timeline in Figure 2.

## Ethical compliance

This study was approved by the Institutional Review Board Committee of Hamamatsu University School of Medicine (16-076) and was performed after obtaining written informed consent. Consent to publish identifiable images was obtained from the patient's parents.

## Variant sequencing

**Figure 3:**
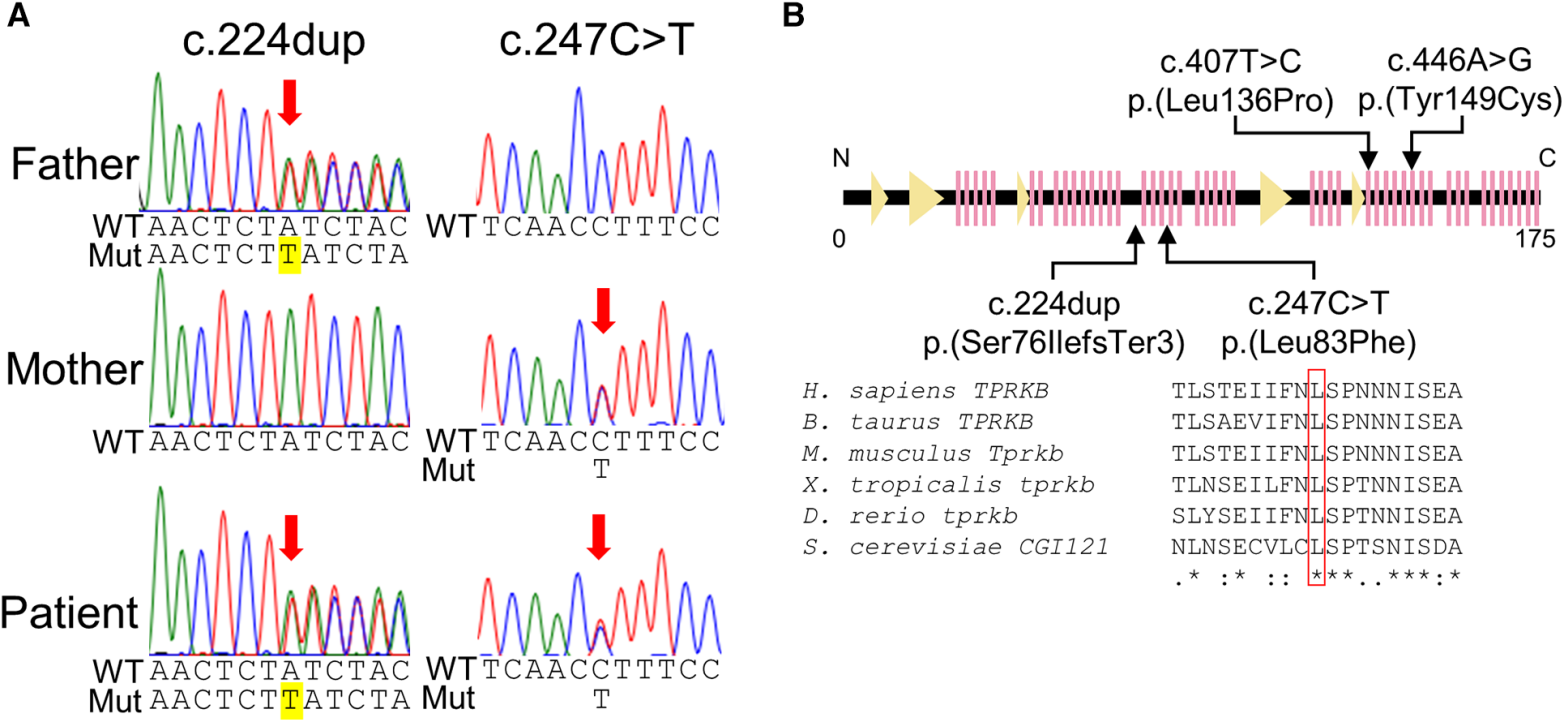
*TPRKB* variants in the patients with Galloway-Mowat syndrome. (**A**) Compound heterozygous variants in *TPRKB*. Sanger sequencing shows a paternal T duplication (yellow) on the left and a maternal missense variant on the right. (**B**) Schematic presentation of the TPRKB protein and location of altered residues. Previously reported TPRKB variants are depicted above, and the variants identified in our case are shown below. The predicted secondary structure modified from the PDB (code 6WQX; https://www.rcsb.org/) is indicated for β-strand and α-helix conformation as triangle and zigzag lines, respectively. Multiple amino acid sequences of TPRKB were aligned using the ClustalW tool (http://www.genome.jp/tools/clustalw).

Genomic DNA was extracted from the blood leukocytes of this family. The patient's DNA for exome sequencing was captured using an xGen Exome Research Panel v2 kit (IDT, Coralville, IA, USA) and sequenced on a NextSeq500 (Illumina, San Diego, CA, USA) with 75-bp paired-end reads. Data processing, variant calling, annotation, and filtering were performed as previously described (11). We identified compound heterozygous variants of *TPRKB* (NM_016058.5), c.224dup, p.(Ser76IlefsTer3) and c.247C > T, p.(Leu83Phe) (Figure 3A). The allele frequencies of the c.224dup and c.247C>T variants were 0.0055% (6/108,604 alleles) and 0.0064% (7/108,604 alleles), respectively, in the ToMMo 54KJPN Allele Frequency Panel (v20230626) (https://jmorp.megabank.tohoku.ac.jp/). The allele frequencies in an East Asian population in the gnomAD v4.0.0 (http://gnomad.broadinstitute.org/) were 0.005039% (2/39,694 alleles) and 0.002520% (1/39,684 alleles), respectively. The c.247C>T, p.(Leu83Phe) variant was predicted to be deleterious using *in silico* pathogenicity prediction tools (Supplementary Table S1), and the Leu83 residue is highly evolutionarily conserved (Figure 3B). The Leu83Phe substitution, like the previously reported TPRKB variants Leu136Pro (α6) and Tyr149Cys (α7), is located at the α helix (α3), a deeply buried position that may affect the structural integrity of the protein (4, 12) (Figure 2B; Supplementary Figure S1). No other likely pathogenic variants were identified among the candidate variants (Supplementary Tables S1, S2). No candidate pathogenic copy number variants were detected using the eXome-hidden Markov model or jNord methods (13, 14). According to the American College of Medical Genetics and Genomics guideline 2015, the c.224dup variant was classified as likely pathogenic and the c.247C>T variant uncertain significance (Supplementary Tables S1, S2). However, since our case had characteristic GAMOS symptoms such as nephrotic proteinuria and central nervous system abnormalities, we concluded that the *TPRKB* variants were likely responsible in this case.

## Discussion

In this study, we describe the case of a patient with novel compound heterozygous *TPRKB* variants. Two individuals with GAMOS with *TPRKB* variants have been previously described, and the clinical manifestations of the three cases of *TPRKB* variants, including our patient, are summarized in Table 1 (4). Microcephaly, global developmental delay, spasticity, and distinctive facial features were common to all three cases. Most patients with GAMOS associated with the KEOPS complex had microcephaly, developmental delay, and distinctive facial features, whereas spasticity was not commonly reported (9/33) (4). This may be because patients with GAMOS with *TPRKB* variants were relatively older children in whom spasticity was evident due to progression of brain atrophy. In our case, proteinuria was diagnosed earlier than in other patients with *TPRKB* variants, possibly because of the loss-of-function variant. The age of onset of proteinuria in patients with TPRKB-related GAMOS was later than that in patients with KEOPS complex-related GAMOS other than TPRKB-related GAMOS (median, three months) (4). Furthermore, the median age of death for GAMOS cases with variants of KEOPS complex-encoding genes other than *TPRKB* was reported to be six months of age. In contrast, one individual with *TPRKB* variant died at 6.8 years of age, and two cases survived at three and six years. TPRKB-related GAMOS may be milder than GAMOS caused by other KEOPS complex-related genes.

**Table 1:** Clinical features of *TPRKB*-associated Galloway-Mowat syndrome**.**

	Braun et al. (4)		This case
	B123	B1144	
Nucleotide change (zygosity)	c.407T>C, (Hom)	c.446A>G, (Hom)	c.224dup, (Het, p) c.247C>T, (Het, m)
Amino acid change	p.Leu136Pro	p.Tyr149Cys	p.Ser76IlefsTer3 p.Leu83Phe
Gender	Male	Male	Male
Ethnic origin	Egyptian	European	Japanese
Age of proteinuria	4.5 years	3.8 years	6 mon
Age of ESRD	6.5 years	No ESRD	No ESRD
Age at Death	6.8 years	Alive with normal renal function (6 years)	Alive with progressive renal dysfunction (3 years)
Biopsy	FSGS	FSGS	Not done
Renal phenotype	SRNS	SRNS (Nephrotic range proteinuria, no edema, serum albumin 3.9 g/L)	Relapsing nephrotic syndrome
Microcephaly	+	+	+
Developmental delay	+	+	+
Spasticity	+	+	+
Distinctive facial features	+	+	+
Brain MRI	Marked brain atrophy with prominent cortical sulci, ventriculomegaly, periventricular white matter demyelination	Pachygyria, periventricular leukomalacia	Marked brain atrophy with delayed myelination and multiple abnormal signals on diffusion-weighted imaging
Others	Deafness	Coordination disorder, ataxia	Hypotonia, pectus excavatum, tapered fingers, esotropia, scoliosis, epilepsy, no bilateral visual evoked potentials

ESRD, end-stage renal disease; FSGS, focal segmental glomerulosclerosis; Het, m, maternal heterozygous; Het, p, paternal heterozygous; Hom, homozygous; MRI, magnetic resonance imaging; SRNS, steroid-resistant nephrotic syndrome.

The most frequently observed brain MRI anomalies in the GAMOS associated with the KEOPS complex include cortical and cerebellar atrophy, gyration abnormalities, and myelination defects (4). Abnormal DWI signals, as in our case, have been reported in patients with GAMOS with pathogenic variants of *TP53RK* and *OSGEP*, which belong to the KEOPS complex (15, 16). MRI of another case of GAMOS attributed to *TP53RK* showed diffuse T2-hypointense signals in the thalamus (17). In animal studies, knockout of *tprkb* in zebrafish larvae recapitulated microcephaly, and *Tprkb* knockout mouse embryos showed a significant reduction in brain size, cortex length, cortex-midbrain midline length, and cortex width (4). Proteomic profiling of fibroblasts from patients with GAMOS caused by *OSGEP* variants revealed upregulation of the P2X7 receptor signaling complex pathway (15). In multiple sclerosis, the sustained activation of P2X7 receptors induces oligodendrocyte death, demyelination, neuroinflammatory processes, and neurodegeneration (18). The activation of P2X7 receptors may be involved in the brain phenotype of KEOPS complex-related GAMOS.

Our patient had NS with repeated relapses concurrently with respiratory infections. Increased edema in patients with congenital NS is especially common during infection (19). Relapsing NS with mild persistent proteinuria and relapses of the nephrotic range associated with upper respiratory tract infections have been described in familial cases of the *NPHS1* variants encoding nephrin, an essential component of the interpodocyte-spanning slit diaphragm (20). The molecular mechanism of NS relapse caused by genetic defects remains to be elucidated and requires further investigation.

The patients with KEOPS complex-related GAMOS have severe renal and neurological symptoms and die in early childhood (4). Kidney complications are the direct cause of most GAMOS deaths, and the patients with GAMOS should be screened regularly for proteinuria and renal function and evaluated closely (21). The genetic diagnosis of GAMOS is useful for family planning, and prenatal and preimplantation testing is available after genetic counseling.

In conclusion, we identified a patient with GAMOS and compound heterozygous *TPRKB* variants who had nephrotic proteinuria, developmental delay, developmental regression, microcephaly, distinctive facial features, skeletal abnormalities, epilepsy, progressive brain atrophy, delayed myelination, T2-hypointense signals in the thalamus, and multiple intracranial abnormal signals. This is the first case of a patient with GAMOS associated with a frameshift variant in *TPRKB*. Further accumulation of cases is necessary to establish the common clinical features in patients with pathogenic *TPRKB* variants.

## Data Availability

The original contributions presented in the study are included in the article/Supplementary Material, further inquiries can be directed to the corresponding author.
