# Supplementary material for: Case Report: Novel compound heterozygous TPRKB variants cause Galloway-Mowat syndrome
Source: Front Pediatr. 2024 Apr 3;12:1360867. doi: 10.3389/fped.2024.1360867 (PMC11018998; doi:10.3389/fped.2024.1360867)
Supplement: Supplementary file 1 [file Table1.docx]

**Table S1. Candidate variants identified by exome sequencing in the patient.**

| Chr | Gene | Variant | Origin | 54KJPN AF | gnomAD global AF | SIFT | PP2 HVAR | CADD phred | M-CAP | Mutation Taster |
| --- | --- | --- | --- | --- | --- | --- | --- | --- | --- | --- |
| 2 | *TPRKB* | NM_016058.5:c.224dup, p.(Ser76IlefsTer3) | Paternal | 0.000055 | 0.000002053 | NA | NA | 32 | NA | NA |
| 2 | *TPRKB* | NM_016058.5:c.247C>T, p.(Leu83Phe) | Maternal | 0.000064 | 6.843e-7 | 0.032 | 0.953 | 26.3 | 0.031 | Deleterious |
| 1 | *KCNN3* | NM_002249.6:c.1411_1412del, p.(Trp471AspfsTer11) | Maternal | − | − | NA | NA | 33 | NA | NA |
| 5 | *FBN2* | NM_001999.4:c.4753C>G, p.(Pro1585Ala) | Paternal | − | − | 0.543 | 0.001 | 15.09 | 0.046 | Benign |
| 5 | *MAP1B* | NM_005909.5:c.2564A>G, p.(Lys855Arg) | Paternal | 0.000009 | 0.000001590 | 0.005 | 0.006 | 22.8 | 0.005 | Benign |
| 9 | *GABBR2* | NM_005458.8:c.2729T>C, p.(Val910Ala) | Maternal | − | 7.540e-7 | 0 | 0.005 | 24.2 | 0.034 | Deleterious |

54KJPN, https://jmorp.megabank.tohoku.ac.jp/; gnomAD v4.0.0 (the Genome Aggregation Database), http://gnomad.broadinstitute.org/; SIFT (Sorting Intolerant From Tolerant), http://sift.jcvi.org/; Polyphen-2 Hum Var, http://genetics.bwh.harvard.edu/pph2/; CADD (Combined Annotation–Dependent Depletion), http://cadd.gs.washington.edu/score; M-CAP (Mendelian Clinically Applicable Pathogenicity), http://bejerano.stanford.edu/mcap/index.html; MutationTaster, http://www.mutationtaster.org/; AF, Allele Frequency; NA, not available.

**Table S2. Variant classification following the** **American College of Medical Genetics and Genomics guideline 2015 (1).**

| **Gene** | **Variant** | **Evidence of pathogenicity** | **Classification** | **OMIM Phenotype (MIM#)** | **Inheritance** |
| --- | --- | --- | --- | --- | --- |
| *TPRKB* | NM_016058.5:c.224dup, p.(Ser76IlefsTer3) | PVS1, PM2 | Likely pathogenic | Galloway-Mowat syndrome 5 (617731) | AR |
| *TPRKB* | NM_016058.5:c.247C>T, p.(Leu83Phe) | PM2, PM3, PP3 | Uncertain significance | Galloway-Mowat syndrome 5 (617731) | AR |
| *KCNN3* | NM_002249.6:c.1411_1412del, p.(Trp471AspfsTer11) | PM2 | Uncertain significance | Zimmermann-Laband syndrome 3 (618658) | AD |
| *FBN2* | NM_001999.4:c.4753C>G, p.(Pro1585Ala) | BP4, PM2 | Likely benign | Contractural arachnodactyly, congenital (121050)  Macular degeneration, early-onset (616118) | AD |
| *MAP1B* | NM_005909.5:c.2564A>G, p.(Lys855Arg) | NA | Uncertain significance | Periventricular nodular heterotopia 9 (618918) | AD |
| *GABBR2* | NM_005458.8:c.2729T>C, p.(Val910Ala) | NA | Uncertain significance | Developmental and epileptic encephalopathy 59 (617904)  Neurodevelopmental disorder with poor language and loss of hand skills (617903) | AD |

AD, Autosomal dominant; AR, Autosomal recessive; NA, not applicable.

PVS1: Null variant (nonsense, frameshift, canonical ±1 or 2 splice sites, initiation codon, single or multiexon deletion) in a gene where LOF is a known mechanism of disease.

PM2: Absent from controls (or at extremely low frequency if recessive) in the Exome Sequencing Project, 1000 Genomes Project, or Exome Aggregation Consortium.

PM3: For recessive disorders, detected in trans with a pathogenic variant.

PP3: Multiple lines of computational evidence support a deleterious effect on the gene or gene product (e.g., conservation, evolutionary, splicing impact).

BP4: Multiple lines of computational evidence suggest no impact on gene or gene product (conservation, evolutionary, splicing impact, etc.).

**Variant interpretation**

Gain of function variants in *KCNN3* cause Zimmermann-Laband syndrome, which is characterized by developmental delay, intellectual disability, coarse face, gingival hyperplasia, and nail hypoplasia/aplasia (2). No loss-of-function variants have been reported to date. Thus, PVS1 did not apply to the p.(Trp471AspfsTer11) variant in *KCNN3*.

**Figure S1**


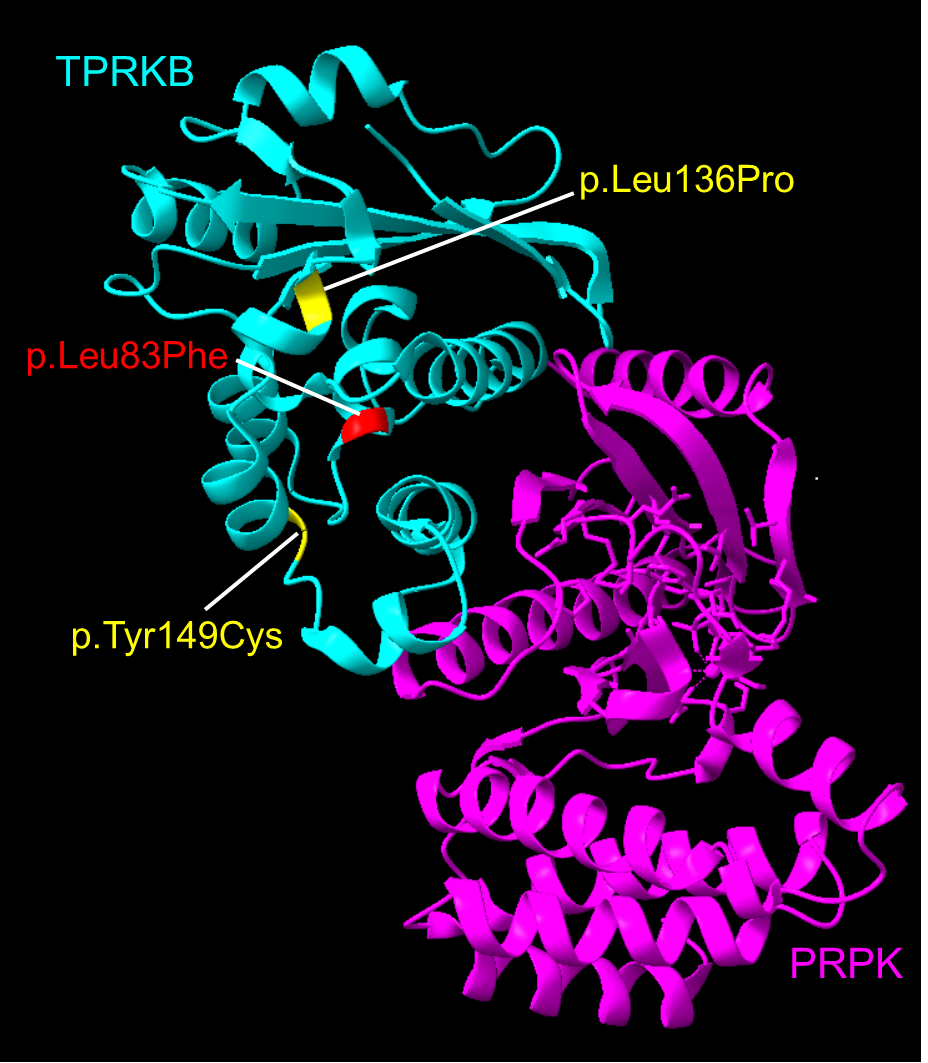


The structure of the TPRKB and PRPK is shown based on a crystal structure using ChimeraX (PDB: ID 6WQX) (TPRKB, cyan; PRPK, purple). The affected residue p.Leu83Phe in TPRKB is indicated in red. Previously reported substituted residues of the TPRKB are indicated in yellow (3).

**REFERENCES**

1. Richards S, Aziz N, Bale S, Bick D, Das S, Gastier-Foster J, et al. Standards and guidelines for the interpretation of sequence variants: a joint consensus recommendation of the American College of Medical Genetics and Genomics and the Association for Molecular Pathology. *Genet Med* (2015) 17(5):405-24. Epub 20150305. doi: 10.1038/gim.2015.30.

2. Bauer CK, Schneeberger PE, Kortüm F, Altmüller J, Santos-Simarro F, Baker L, et al. Gain-of-Function Mutations in KCNN3 Encoding the Small-Conductance Ca2+-Activated K+ Channel SK3 Cause Zimmermann-Laband Syndrome. *Am J Hum Genet* (2019) 104(6):1139-1157. Epub 20190530. doi: 10.1016/j.ajhg.2019.04.012.

3. Braun DA, Rao J, Mollet G, Schapiro D, Daugeron MC, Tan W, et al. Mutations in KEOPS-complex genes cause nephrotic syndrome with primary microcephaly. *Nat Genet* (2017) 49(10):1529-1538. Epub 20170814. doi: 10.1038/ng.3933.
